# Dose response relationship of cumulative anticholinergic exposure with incident dementia: validation study of Korean anticholinergic burden scale

**DOI:** 10.1186/s12877-020-01671-z

**Published:** 2020-07-29

**Authors:** Yewon Suh, Young-Mi Ah, Euna Han, Kwanghee Jun, Sunghee Hwang, Kyung Hee Choi, Ju-Yeun Lee

**Affiliations:** 1 College of Pharmacy and Research Institute of Pharmaceutical Sciences, Seoul National University, 1 Gwanak-ro, Gwanak-gu, Seoul, 08826 Republic of Korea; 2 Department of Pharmacy, Seoul National University Bundang Hospital, 82, Gumi-ro 173 Beon-gil, Bundang-gu, Seongnam-si, Gyeonggi-do 13620 Republic of Korea; 3 College of Pharmacy, Yeungnam University, 280 Daehak-ro, Gyeongsan-si, Gyeongsangbuk-do 38541 Republic of Korea; 4 College of Pharmacy, Yonsei Institute for Pharmaceutical Research, Yonsei University, 85 Songdogwahak-ro, Yeonsu-gu, Incheon, 21983 Republic of Korea; 5 College of Pharmacy and Institute of Pharmaceutical Science and Technology, Hanyang University, 55 Hanyangdeahak-ro, Sangnok-gu, Ansan-si, Gyeonggi-do 15588 Republic of Korea; 6 College of Pharmacy, Sunchon National University, 255 Jungang-ro, Suncheon-si, Jeollanam-do 57922 Republic of Korea

**Keywords:** Aged, Anticholinergic agents, Anticholinergic burden scale, Dementia

## Abstract

**Background:**

The dose response relationship of nine-year cumulative anticholinergic exposure and dementia onset was investigated using the Korean version anticholinergic burden scale (KABS) in comparison with the Anticholinergic Cognitive Burden Scale (ACB). We also examined the effect of weak anticholinergics in the prediction of dementia.

**Methods:**

A retrospective case-control study was conducted comprising 86,576 patients after 1:2 propensity score matching using the longitudinal national claims database. For cumulative anticholinergic burden estimation, average daily anticholinergic burden score during the 9 years prior to dementia onset was calculated using KABS and ACB and categorized as minimal, < 0.25; low, 0.25–1; intermediate, 1–2; and high, ≥ 2. Adjusted odds ratio (aOR) between cumulative anticholinergic burden and incident dementia was estimated.

**Results:**

Patients with high exposure according to KABS and ACB comprised 3.2 and 3.4% of the dementia cohort and 2.1 and 2.8% of the non-dementia cohort, respectively. Dose-response relationships were observed between anticholinergic burden and incident dementia. After adjusting covariates, compared with minimal exposure, patients with high exposure according to KABS and ACB had a significantly higher risk for incident dementia with aOR of 1.71 (95% confidence interval (CI) 1.55–1.87) and 1.22 (CI 1.12–1.33), respectively. With the exclusion of weak anticholinergics, the association became stronger, i.e., 1.41 (CI 1.14–1.75) with ACB whereas the association became slightly weaker with KABS, i.e., 1.60 (CI 1.38–1.86).

**Conclusion:**

This study confirmed the dose response relationship for cumulative anticholinergic burden measured using the Korean specific anticholinergic burden scale with incident dementia.

## Background

Dementia, especially Alzheimer’s disease, is caused by a combination of ageing, genetic, health, environmental, and lifestyle factors. There is a great deal of interest in identifying the modifiable risk factors of cognitive decline and dementia [1]. Previous studies suggested that the use of medications such as anticholinergics is related to worsening of cognitive functions or incidence of dementia [2, 3]. Several tools for measuring anticholinergic burden, the summed effect of multiple medications with various anticholinergic potency, have been developed to predict the potential adverse outcome of anticholinergic use. The Anticholinergic Cognitive Burden Scale (ACB), Anticholinergic Drug Scale (ADS), and Anticholinergic Risk Scale (ARS) are the three most popular tools [4]. The advantage of these tools over lists of strong anticholinergics might be that they allow the summation of unrecognized medications with weak anticholinergic effects as well as strong anticholinergics for measuring the anticholinergic burden [5].

However, previously developed tools cannot be directly applied to practice in other countries because medication availability differs considerably, and considerable inconsistencies exist among the tools regarding the listed medications and their anticholinergic potency scores. Therefore, the Korean version anticholinergic burden scale (KABS) was developed using the Delphi methods after reviewing previous scales for the medications having discordant scores among scales and for the newly added medications that have been available in Korea but have not been reviewed [6].

The longitudinal effect of anticholinergics on incident dementia or cognitive impairment has been investigated in several studies with controversial results [2, 7–12]. Some studies defined the exposure of anticholinergics with the continued or cumulative use of strong anticholinergics without considering the anticholinergic score [2, 7, 8, 11]. However, only a few studies used the anticholinergic burden scale which includes both the strong and the weak anticholinergics with the scoring system. Richardson et al. [9] examined the association between anticholinergic burden assessed with the ACB scale and dementia risk using a nested case-control study in the UK. Hsu et al. [10] showed a dose response relationship of anticholinergic burden measured with ARS, ACB, and Drug Burden Index-Anticholinergics with incident dementia as one of the adverse outcomes using Taiwan’s National Health Insurance data.

We hypothesized that a greater cumulative anticholinergic exposure measured using the burden scale increases the risk of incident dementia and that the KABS, consensus-driven, Korea specific anticholinergic burden scale, will outperform the tool developed from other countries in predicting the risk of dementia in the Korean population. We aimed to evaluate the validity of KABS in comparison with that of the ACB scale by investigating the association between nine-year cumulative anticholinergic exposure and dementia using the longitudinal nationally representative cohort comprising the elderly population. In addition, we also aimed to investigate whether the inclusion of weak anticholinergics in measuring anticholinergic burden influences the prediction of dementia.

## Method

### Study population and database

A longitudinal database was used for this case control study, the Korea National Health Insurance Service Senior Cohort database (2002–2013) that was developed and provided by the Korea National Health Insurance Service, a single and mandatory national insurance service. This database comprised 558,147 patients over 60 years, representing 10% of the Korean senior population. Its representativeness was supported by the results of a study comparing this database with population statistics based on resident registration, Statistics Korea’s data of December 2013 [13].

Older adults with no diagnosis of dementia during the prior years of 2002–2011 and alive as on January 1, 2013, were initially selected from the Korea National Health Insurance Service Senior Cohort database. Among them, patients who were diagnosed with dementia (either vascular or Alzheimer’s or both; ICD-10 codes: F00, F01, F02, F03, F051, G30, and G311) in 2012–2013 were selected. Index dates were defined as the earliest date of claims with the diagnostic code for dementia. Control cohorts were selected among the patients without dementia after propensity score matching. Details are described in statistical analysis.

### Measure of anticholinergic burden

**Fig. 1:**
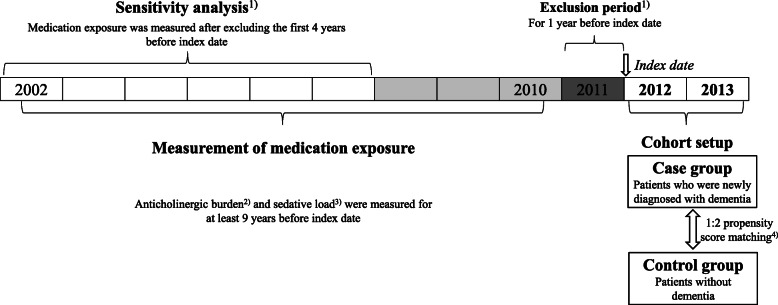
The layout of research design. ^1)^ In order to minimize the protopathic bias, the first year before index date was excluded. Also, sensitivity analysis was done after excluding the first 4 years before index date. ^2)^ The Korean version anticholinergic burden scale (KABS) and the Anticholinergic Cognitive Burden Scale (ACB) were used as the standard of anticholinergic burden score. ^3)^ For measuring the sedative load, the sedative load model was used; score 2 for primary sedative agents and score 1 for medications having sedation as a prominent side effects. ^4)^ Matching variables: age, sex, and baseline co-morbid diseases including hypertension, dyslipidemia, heart failure, atrial fibrillation, ischemic heart disease, diabetes mellitus, cerebrovascular disease, Parkinson’s disease, depression, anxiety, insomnia, and alcohol disease

Exposure to anticholinergic drugs was measured during the nine prior years starting 10 years before the index date and ending 1 year before the index date. Drug exposure in the recent one-year period was excluded to minimize the protopathic bias. Nine-year cumulative drug exposure was measured in all patients (Fig. 1). The anticholinergic burden for each patient was assessed using KABS and ACB (Additional file 1). Both scales assigned 1 for mild, 2 for moderate, and 3 for strong anticholinergics. First, the standard daily dose was calculated with the prescribed dose for each medication divided by the defined daily dose (DDD) of the World Health Organization. Then, we summed the standard daily dose for anticholinergic drugs after multiplying the assigned anticholinergic score during the period of drug exposure. This method was modified from that suggested by Gray et al. [8] with multiplying the anticholinergic score assigned by each anticholinergic burden scale. We finally calculated the average daily anticholinergic burden score during the drug exposure period. We categorized the anticholinergic burden according to daily average score as follows: < 0.25, minimal exposure; 0.25–1, low exposure; 1–2, intermediate exposure; and ≥ 2, high exposure. We further investigated the anticholinergic drugs most contributing to high exposure prior to the index date in patients with dementia according to KABS or ACB.

### Covariates

As confounders that might affect the onset of dementia, we selected baseline comorbid diseases such as hypertension, dyslipidemia, heart failure, atrial fibrillation, ischemic heart disease, diabetes mellitus, cerebrovascular disease, Parkinson’s disease, depression, anxiety, schizophrenia, bipolar disorder, insomnia, alcohol disease, obesity, substance abuse, and tobacco dependence and use.

These comorbid diseases were identified when there were two or more claims with the relevant diagnostic code (Additional file 2) before January 1, 2012. For covariates, the sedative load during the same period was calculated. We used the sedative load model for measuring the sedative load, which assigned 2 for the primary sedative and 1 for medications having sedation as a prominent side effect. We excluded the medications that were assigned 1 or a higher anticholinergic score by each scale for measuring the sedative load (Additional file 3).

### Statistical analysis

To reduce the effect of confounding factors, control cohorts were selected using the propensity score matching method with the ratio of 1:2 using variables including age, sex, and baseline comorbid diseases that are known to cause of dementia including hypertension, dyslipidemia, heart failure, atrial fibrillation, ischemic heart disease, diabetes mellitus, cerebrovascular disease, Parkinson’s disease, depression, anxiety, insomnia, and alcohol disease. The matching was performed using a greedy algorithm with a caliper width equal to 0.2 standard deviations of the logit of the propensity score. The measured anticholinergic burden by both scales was described and compared between the case and control cohorts. We used Chi-square test for comparison of categorical variables for baseline characteristics. Multivariate logistic regression analysis was performed to evaluate the independent association between the anticholinergic burden and the incidence of dementia to adjust for the covariates including age, sex, the baseline co-morbid diseases, and sedative load. Adjusted odds ratios (aOR) with 95% confidence intervals (CI) are reported.

In the secondary analysis, we also investigated the impact of weak anticholinergic agents on each scale after excluding them. To minimize reverse causation bias, the association between incident dementia and the cumulative anticholinergic burden during the 5–10 years prior to the index date after excluding the most recent 4 years was analyzed as part of the sensitivity analysis. Data management and statistical analysis were performed using SAS version 9.3 (SAS Institute, Inc., Cary, NC, USA).

## Results

### Population characteristics

**Fig. 2:**
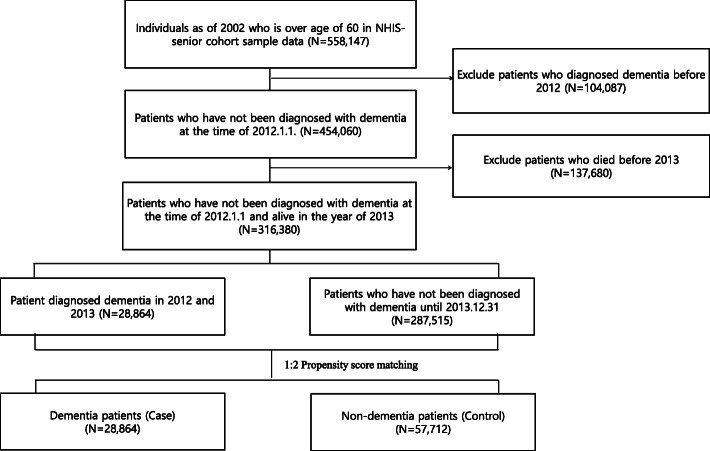
Flow chart for selecting patients. NHIS; National Health Insurance Service

Among the 558,147 adults aged more than 60 as of 2002 included in the sample senior cohort, 104,087 patients and 137,680 patients were excluded because they were diagnosed with dementia before the year 2012 and because they had not survived until the year 2013, respectively. The total sampled population included 28,864 patients who were diagnosed with dementia between 2012 and 2013. After propensity score matching, 57,712 patients without dementia were identified as the control and in total 86,576 patients were included for the analysis (Fig. 2).

The median age at the time of dementia diagnosis was 79 (inter-quartile range (IQR), 75–83) and women accounted for 69.3%. As the comorbid condition, 79, 61, 49, 46, 45, 39, 35 and 30% of the patients had hypertension, dyslipidemia, diabetes, anxiety, cerebrovascular disease, insomnia, ischemic heart disease, and depression, respectively (Table 1).

**Table 1:** Demographic data of patients with and without dementia before and after propensity score matching

	Before matching			After matching		
	Non-Dementia (***N*** = 287,515)	Dementia (***N*** = 28,865)	***p***-value	Non-Dementia (***N*** = 57,712)	Dementia (***N*** = 28,864)	***p***-value
**Age, median (IQR)**	76 (73 ~ 80)	79 (75 ~ 93)		79 (75–83)	79 (75–83)	
70–80 years	214,874 (74.7)	15,894 (55.1)	< 0.001	31,621 (54.8)	15,894 (55.1)	0.400
80–90 years	65,342 (22.7)	11,041 (38.2)		22,098 (38.3)	11,041 (38.3)	
≥ 90 years	7,299 (2.6)	1,930 (6.7)		3,993 (6.9)	1,929 (6.7)	
**Sex**, female	170,007 (59.1)	20,005 (69.3)	< 0.001	40,338 (69.9)	20,004 (69.3)	0.744
**Co-morbid disease**						
Hypertension	203,036 (70.6)	22,848 (79.2)	< 0.001	46,059 (79.8)	22,848 (79.2)	0.371
Dyslipidemia	157,248 (54.7)	17,545 (60.8)	< 0.001	35,262 (61.1)	17,545 (60.8)	0.761
Ischemic heart disease	81,060 (28.2)	10,123 (35.1)	< 0.001	20,201 (35.0)	10,123 (35.1)	0.843
Heart failure	41,404 (14.4)	6,266 (21.7)	< 0.001	12,353 (21.4)	6,266 (21.7)	0.305
Atrial fibrillation	10,614 (3.7)	1,491 (5.2)	< 0.001	2,603 (4.5)	1,491 (5.2)	< 0.001
Diabetes Mellitus	113,939 (39.6)	14,113 (48.9)	< 0.001	28,084 (48.7)	14,113 (48.9)	0.519
Cerebrovascular disease	76,776 (26.7)	12,863 (44.6)	< 0.001	25,917 (44.9)	12,863 (44.6)	0.338
Depression	52,179 (18.2)	8,555 (29.6)	< 0.001	16,923 (29.3)	8,555 (29.6)	0.336
Bipolar disorder	2,853 (1.0)	540 (1.9)	< 0.001	889 (1.5)	540 (1.9)	0.003
Schizophrenia	1,662 (0.6)	354 (1.2)	< 0.001	460 (0.8)	354 (1.2)	< 0.001
Anxiety	97,503 (33.9)	13,385 (46.4)	< 0.001	26,680 (46.2)	13,385 (46.4)	0.691
Insomnia	78,382 (27.3)	11,320 (39.2)	< 0.001	22,611 (39.2)	11,320 (39.2)	0.911
Substance abuse	1,663 (0.6)	307 (1.1)	< 0.001	498 (0.9)	307 (1.1)	0.004
Tobacco dependence and Tobacco use	113 (0.0)	15 (0.02)	0.308	22 (0.04)	15 (0.02)	0.353
Parkinson’s disease	4,893 (1.7)	1,371 (4.8)	< 0.001	2,305 (4.0)	1,371 (4.8)	< 0.001
Obesity	472 (0.2)	42 (0.2)	0.453	97 (0.2)	42 (0.2)	0.434
Alcohol disease	1,566 (0.5)	280 (1.0)	< 0.001	467 (0.8)	280 (1.0)	0.016

*IQR* Inter-quartile range

### Prevalence of anticholinergic burden

During the 2–10 years before the index date, 46.2% of patients with dementia and 50.7% of patients without dementia were exposed minimally to anticholinergics (average daily KABS score <  0.25). Fewer patients were identified as having intermediate anticholinergic exposure using KABS compared to that using ACB in both cohorts with dementia (10.6 and 13.7%, *p* <  0.01, respectively) and without dementia (8.9 and 12.9%, *p* < 0.01). Proportions of patients with high exposure were 2.1% (for the KABS) and 2.8% (for the ACB) in the cohort without dementia and 3.2% (for the KABS) and 3.4% (for the ACB) in the cohort with dementia (Table 2).

**Table 2:** Prevalence of anticholinergic burden score measured using KABS and ACB with and without including weak anticholinergics

	Non-dementia (*N* = 57,712)				Dementia (*N* = 28,864)			
	KABS	KABS-1	ACB	ACB-1	KABS	KABS-1	ACB	ACB-1
Anticholinergic exposure during 2–10 year before index year								
Minimal (< 0.25)	29,280 (50.7%)	38,227 (66.2%)	23,890 (41.4%)	48,142 (83.4%)	13,347 (46.2%)	17,739 (61.5%)	11,502 (39.9%)	23,031 (79.8%)
Low (0.25–1)	22,060 (38.2%)	16,555 (28.7%)	24,785 (43.0%)	8,176 (14.2%)	11,556 (40.0%)	9,167 (31.8%)	12,433 (43.1%)	4,854 (16.8%)
Intermediate (1–2)	5,136 (8.9%)	2,488 (4.3%)	7,447 (12.9%)	1,188 (2.1%)	3,045 (10.6%)	1,630 (5.6%)	3,954 (13.7%)	830 (2.9%)
High (≥2)	1236 (2.1%)	442 (0.8%)	1590 (2.8%)	206 (0.4%)	916 (3.2%)	328 (1.1%)	975 (3.4%)	149 (0.5%)
Anticholinergic exposure during 5–10 year before index year								
Minimal (< 0.25)	32,424 (56.2%)	40,272 (69.8%)	27,297 (47.3%)	50,009 (86.7%)	15,283 (53.0%)	19,174 (66.4%)	13,566 (47.0%)	24,226 (83.9%)
Low (0.25–1)	19,959 (34.6%)	14,903 (25.8%)	22,432 (38.9%)	6,662 (11.5%)	10,389 (36.0%)	8,093 (28.0%)	11,105 (38.5%)	3,937 (13.6%)
Intermediate (1–2)	4,263 (7.4%)	2128 (3.7%)	6,539 (11.3%)	862 (1.5%)	2,482 (8.6%)	1,333 (4.6%)	3,372 (11.7%)	588 (2.0%)
High (≥2)	1,066 (1.9%)	409 (0.7%)	1,444 (2.5%)	179 (0.3%)	710 (2.5%)	264 (0.9%)	821 (2.8%)	113 (0.4%)

*KABS* Korean anticholinergic burden scale, *KABS-1* Korean anticholinergic burden scale without medications of score 1, *ACB* Anticholinergic cognitive burden, *ACB-1* Anticholinergic cognitive burden without medications of score 1

### Major anticholinergic drugs

The top 20 anticholinergic drugs that most contributed to high exposure prior to the index date in patients with dementia according to KABS and ACB are presented in Table 3. Cimetidine, dimenhydrinate, chlorpheniramine, furosemide, and diazepam contributed the most to the total KABS score, while dimenhydrinate, hydrochlorothiazide, isosorbide, chlorpheniramine, and nifedipine contributed most to the total ACB score in patients with dementia with high exposure. Drugs with a KABS score of 3, 2, and 1 contributed 47.4, 19.3, and 33.3%, respectively, to the cumulative amount of the KABS score, whereas those with an ACB score of 3, 2, and 1 contributed 41.4, 1.8, and 56.7%, respectively, to the cumulative amount of the ACB score in dementia patients with high exposure.

**Table 3:** Top 20 medications that contributed to the anticholinergic burden score in dementia patients with high exposure prior to 2–10 years of dementia according to the scales

Drug	Total (***N*** = 1,324)			KABS (***N*** = 916)		ACB (***N*** = 975)	
	Number of patients (%)		Average DDD per patient per year	score	Contribution to high exposure (%)	score	Contribution to high exposure (%)
chlorpheniramine^a, b^	1,297	(98.0)	17	3	5.8	3	5.2
tramadol^a^	1,262	(95.3)	9.2	2	2.3	0	0
cimetidine^a, b^	1,260	(95.2)	46.7	2	11.6	1	4.0
diazepam^a, b^	1,198	(90.5))	34.8	1	4.2	1	3.4
ranitidine^a, b^	1,150	(86.9)	28.6	1	3.1	1	3.0
hydrochlorothiazide^b^	974	(73.6)	80.2	0	0	1	10.1
alprazolam^a, b^	921	(69.6)	32.5	1	4.1	1	3.2
dimenhydrinate^a, b^	869	(65.6)	33.3	3	11.3	3	11.7
amitriptyline^a, b^	727	(54.9)	10.9	3	3.8	3	3.4
theophylline^a, b^	664	(50.2)	13.8	1	1.5	1	1.5
furosemide^a, b^	606	(45.8)	39.5	1	4.3	1	5.0
atenolol^b^	549	(41.5)	33	0	0	1	4.3
lorazepam^a^	515	(38.9)	11.9	1	1.5	0	0
nifedipine^b^	459	(34.7)	38	0	0	1	5.2
triazolam^a^	454	(34.3)	15.8	1	2.1	0	0
octylonium bromide^a^	451	(34.1)	4.5	3	1.8	0	0
tolterodine^a, b^	355	(26.8)	7.9	3	2.7	3	2.8
propiverine^a, b^	342	(25.8)	6.3	3	2.2	3	2.2
isosorbide^b^	290	(21.9)	40.2	0	0	1	5.4
doxazosin^b^	263	(19.9)	18.1	0	0	1	2.4
digoxin^b^	215	(16.2)	13.3	1	1.2	1	1.7
paroxetine^a, b^	209	(15.8)	9.3	2	2.1	3	3.5
quinupramine^a^	188	(14.2)	5.1	3	2.2	0	0
solifenacin^a, b^	174	(13.1)	6.1	3	2.1	3	2.3
levodopa and decarboxylase inhibitor^a^	166	(12.5)	12.6	1	1.8	0	0
beztropine^b^	77	(5.8)	3.6	3	1.4	3	1.3
amantadine^a^	76	(5.7)	6.3	2	1.8	2	1.1

*DDD* Defined daily dose, *KABS* Korean Anticholinergic Burden Scale, *ACB* Anticholinergic Cognitive Burden

^a^Top 20 medications to contribute to anticholinergic burden in high exposure group with KABS

^b^Top 20 medications to contribute to anticholinergic burden in high exposure group with ACB

### Association between anticholinergic burden and incident dementia

There were significant associations between incident dementia and nine-year cumulative dose adjusted anticholinergic burden of low, intermediate, and high exposure compared with that of minimal exposure assessed by KABS with corresponding aOR of 1.21 (95% CI, 1.17–1.25), 1.39 (95% CI, 1.31–1.46), and 1.71 (95% CI, 1.55–1.87), respectively. A similar association was observed when the anticholinergic burden was measured using ACB but corresponding aORs were lower than those measured using KABS. A high cumulative anticholinergic burden measured using the ACB was significantly associated with dementia incidence with an aOR of 1.22 (95% CI, 1.12–1.33) (Table 4).

**Table 4:** Adjusted odds ratios for incident dementia according to anticholinergic burden measured using KABS and ACB with and without weak anticholinergics

	Including medications with score 1		Excluding medications with score 1	
	KABS aOR^a^ (95% CI)	ACB aOR^a^ (95% CI)	KABS-1 aOR^a^ (95% CI)	ACB-1 aOR^a^ (95% CI)
Anticholinergic exposure during 2–10 years before index year				
Minimal (< 0.25)	reference	reference	reference	reference
Low (0.25–1)	1.21 (1.17–1.25)	1.06 (1.04–1.10)	1.23 (1.19–1.28)	1.24 (1.19–1.29)
Intermediate (1–2)	1.39 (1.31–1.46)	1.10 (1.04–1.15)	1.45 (1.36–1.56)	1.42 (1.30–1.56)
High (≥2)	1.71 (1.55–1.87)	1.22 (1.12–1.33)	1.60 (1.38–1.86)	1.41 (1.14–1.75)
Anticholinergic exposure during 5–10 years before index year				
Minimal (< 0.25)	reference	reference	reference	reference
Low (0.25–1)	1.14 (1.10–1.18)	1.00 (0.96–1.03)	1.17 (1.13–1.21)	1.22 (1.17–1.28)
Intermediate (1–2)	1.27 (1.20–1.35)	1.02 (0.97–1.08)	1.33 (1.24–1.44)	1.38 (1.24–1.54)
High (≥2)	1.44 (1.30–1.59)	1.09 (0.99–1.19)	1.35 (1.15–1.58)	1.23 (0.97–1.56)

*aOR* Adjusted odds ratio, *CI* Confidence interval, *KABS* Korean anticholinergic burden scale, *ACB* Anticholinergic cognitive burden, *KABS-1* Korean anticholinergic burden scale without medications of score 1, *ACB-1* Anticholinergic cognitive burden without medications of score 1

^a^Adjusted for age, sex, sedative load and comorbid diseases (hypertension, dyslipidemia, heart failure, atrial fibrillation, ischemic heart disease, diabetes mellitus, cerebrovascular disease, Parkinson’s disease, depression, anxiety, schizophrenia, bipolar disorder, insomnia, alcohol disease, obesity, substance abuse, and tobacco dependence and use)

When the anticholinergic burden was measured after excluding weak anticholinergics (score = 1), the proportion of patients with low to high anticholinergic exposure was significantly reduced, and this reduction was much greater using the ACB scale versus that using the KABS. The effect size of the association with incident dementia was similar when exposure was measured using KABS without regard to the inclusion of weak anticholinergics. The KABS scale with weak anticholinergics showed slightly greater association than that without weak anticholinergics regarding high exposure (aOR 1.71 [1.55–1.87] vs. 1.60 [1.38–1.86]). However, the odds ratios for low, intermediate, and high vs. minimal cumulative exposure increased from 1.06–1.22 to 1.24–1.41 when the anticholinergic burden was measured using ACB excluding weak anticholinergics. No substantial changes were found in the association of anticholinergic exposure with the incidence of dementia after measuring drug exposure 5–10 years prior to the index date. This led to small reductions in the associations.

## Discussion

This study confirmed that cumulative anticholinergic exposure is associated with the incidence of dementia. Even though the direct comparison is not possible owing to different study designs and methods, this finding supports the results of previous studies [8, 9, 11].

Additionally, we showed the significant dose-response relationship of anticholinergic burden measured using KABS, compared to that using ACB, with incident dementia which suggests KABS, the anticholinergic burden scale specific to the Korean population, can be used as a validated tool to predict the risk of dementia, at least among the older Korean population.

While most previous studies measured anticholinergic burden with the use of strong anticholinergics [2, 7, 8, 11], we measured anticholinergic burden using the scales that ranked the anticholinergic potency as a score for each agent including weak anticholinergics. According to our findings, compared with minimal anticholinergic use, an average daily anticholinergic score of 2 or higher was associated with a 1.71-fold increase in the odds of incident dementia.

A retrospective cohort study which examined the anticholinergic burden with incident dementia as one of the adverse clinical outcomes using the Taiwan national claims database [10] suggested ACB shows good dose response relationship with incident dementia. Compared with the present study, that study showed greater associations (aOR 3.13–10.01 in 65–74 years, aOR 2.76–7.44 in 75–84 years). However, they did not exclude the anticholinergic used for the treatment of prodromal symptoms of dementia. A large community-based longitudinal cohort study conducted by Gray et al. [8] showed that strong anticholinergics used for more than 3 years with mean 7.3 ± 4.8 years of follow up period was associated with increased risk of dementia (hazard ratio, 1.54 (95% CI, 1.21–1.96).

We also showed the negative impact of including weak anticholinergics (score = 1) on the magnitude of association with incident dementia when the ACB scale was used. These results were consistent with the findings from previous studies [9, 11], where no relationship or no dose response relationship were observed between exposure to a greater number of drugs with an ACB score 1 and incident dementia. However, this finding was not observed when the burden was measured using KABS. This might be explained by the difference in medication lists of weak anticholinergics between KABS and ACB. In the ACB scale, cardiovascular agents such as hydrochlorothiazide, atenolol, nifedipine, and isosorbide are included as weak anticholinergics, which were determined as score 0 in the KABS by expert consensus after a comprehensive review of other existing scales [6]. This finding was consistent with that from a previous study that showed a slightly negative association of cardiovascular anticholinergic agents with dementia [9]. Based on these findings, the recommendation of reduced use of these cardiovascular agents to reduce the anticholinergic burden to prevent dementia should be deferred until further research. The significant differences in the prevalence and the association with clinical outcome of anticholinergic exposure according to the scales were consistent with the results from a previous study [14].

A sensitivity analysis that investigated the association after excluding most recent 4 years prior to the index date to minimize the effect of using anticholinergics for the prodromal syndrome of dementia showed the identical trend in the effect of anticholinergic burden to the onset of dementia. Thus, we confirmed the robustness of the correlation between anticholinergic burden and dementia onset. Additionally, this result is similar to that from a previous case-control study by Richardson and colleagues [9]. They categorized exposure using the cumulative DDD and average ACB score. The daily average ACB score of 2 during the prior 5–10 years in our study corresponds to 1460 DDD of drugs with a score of 3. They showed weak associations with 1460 DDD of drugs with a score of 1, which corresponds to the daily average ACB score of 0.7.

The strengths of this study are 1) we took into account the prescribed dosage and potency of anticholinergics when measuring anticholinergic burden unlike in most previous studies, 2) we analyzed the long-term drug exposure in all participants of a nationally representative large sample with the consideration of reverse causation bias, 3) we considered sedative load other than anticholinergics as a covariate to adjust for the effect of sedatives and medications with sedation as a prominent side effects on incidence of dementia, and 4) we tested the performance of the ACB and KABS scales for the prediction of dementia with or without weak anticholinergics. This was the first attempt to test the influence of including weak anticholinergics in measuring burden using these scales on the prediction of the incidence of dementia.

There are several limitations to consider when interpreting the results of the present study. First, although we carefully addressed the confounding factors associated with the incidence of dementia with propensity matching and multivariate analysis, we could not consider the other confounding factors such as smoking, alcohol consumption, family history, and educational level that might contribute to the incidence of dementia owing to the nature of data analyzed. Second, we could not consider taking non-prescription anticholinergics such as first-generation antihistamines. Third, the diagnosis of a disease might not be correct owing to the nature of claims data. To reduce the possibilities of misdiagnosis, patients were considered as having the corresponding diseases when the diagnostic codes were presented at least twice. Additionally, the diagnosis of dementia might be missed if the patients did not visit healthcare facilities for the symptoms.

Considering the robust associations between high anticholinergic exposure and incident dementia from the present study consistent with previous studies, it is necessary to provide physicians and clinical pharmacists with a calculated anticholinergic burden score for specific patients. This process has been proven to help reduce the anticholinergic burden [15].

## Conclusion

This study confirmed the findings of previous studies by showing the dose response relationship for cumulative anticholinergic burden measured using the KABS, the anticholinergic burden scale specific for Korean populations, with incident dementia.

## Supplementary information

**Additional file 1.** Anticholinergic medication list.

**Additional file 2.** The ICD-10 codes used for analysis.

**Additional file 3.** List of medication with sedation as a prominent side effects.

## Data Availability

The dataset supporting the conclusions of this article is available from the Korea National Health Insurance Service (KNHIS) Data Sharing Service homepage (https://nhiss.nhis.or.kr/bd/ab/bdaba001cv.do) but restrictions apply to the availability of these data. The KNHIS, the data provider, requires all involved researchers to pledge not to share, release, or review the data with other entities.

## Abbreviations

ACB: Anticholinergic Cognitive Burden scale
ADS: Anticholinergic Drug Scale
aOR: Adjusted odds ratio
ARS: Anticholinergic Risk Scale
CI: Confidence Interval
DDD: Defined Daily Dose
ICD: International Classification of Diseases
IQR: Inter-quartile range
KABS: Korean version Anticholinergic Burden Scale
