# Supplementary material for: Dose response relationship of cumulative anticholinergic exposure with incident dementia: validation study of Korean anticholinergic burden scale
Source: BMC Geriatr. 2020 Jul 29;20:265. doi: 10.1186/s12877-020-01671-z (PMC7391507; doi:10.1186/s12877-020-01671-z)
Supplement: Supplementary file 1 — Additional file 1. Anticholinergic medication list. [file 12877_2020_1671_MOESM1_ESM.docx]

Anticholinergic medication list

| **Medications** | **ACB** | **K-ABS** |
| --- | --- | --- |
| ***Analgesics*** *(N=30)* |  |  |
| Acetaminophen/Dichloralphenazone/Isometheptene, Acetaminophen, Aspirin, Buprenorphine, Celecoxib, Diclofenac, Etodolac, Hydromorphone, Ibuprofen, Ketoprofen, Ketorolac, Meloxicam, Nabumetone, Naproxen, Naratriptan, Pentazocine, Piroxicam, Salsalate, Sulindac, Sumatriptan, Tiaprofenic, Zolmitriptan | - | 0 |
| Hydrocodone, Oxycodone | 0 | 1 |
| Codeine, Fentanyl, Morphine | 1 | 1 |
| Nefopam, Pethidine | 2 | 2 |
| Tramadol | - | 2 |
| ***Anti-dementia drugs*** *(N=5)* |  |  |
| Donepezil, Galantamine, Ginkgo, Memantine, Rivastigmine | - | 0 |
| ***Antidepressants*** *(N=20)* |  |  |
| Duloxetine, Moclobemide, Sertraline, Tianeptine^†^ | - | 0 |
| Fluoxetine | - | 1 |
| Bupropion, Citalopram, Dexvenlafaxine, Escitalopram, Fluvoxamine, Mirtazapine, Trazodone, Venlafaxine | 1 | 1 |
| Paroxetine | 3 | 2 |
| Amitriptyline, Amoxapine, Clomipramine, Imipramine, Nortriptyline | 3 | 3 |
| ***Antiepileptics*** *(N=10)* |  |  |
| Levetiracetam | - | 0 |
| Gabapentin, Lamotrigine, Phenobarbital, Phenytoin, Primidone, Topiramate, Valproic acid | - | 0 |
| Carbamazepine | 2 | 1 |
| Oxcarbazepine | 2 | 2 |
| ***Antihistamines*** *(N=31)* |  |  |
| Azelastine^†^, Bepotastine^†^, Ebastine^†^, Epinastine^†^, Fexofenadine, Mizolastine^†^, Olopatadine^†^, Oxatomide^†^ | - | 0 |
| Emedastine^†^, Ketotifen^†^ | - | 1 |
| Cetirizine, Desloratadine, Levocetirizine, Loratadine, Trimeprazine;Alimemazine | 1 | 1 |
| Triprolidine | - | 2 |
| Cyproheptadine | 2 | 2 |
| Dexbrompheniramine, Homochlorcyclizine^†^, Mequitazine^†^, Pheniramine^†^, Piprinhydrinate^†^ ; diphenylpyraline | - | 3 |
| Brompheniramine, Carbinoxamine, Chlorpheniramine, Clemastine, Dexchlorpheniramine, Diphenhydramine, Doxylamine, Hydroxyzine, Pyrilamine ; Mepiramine | 3 | 3 |
| ***Anti-infectives*** *(N=47)* |  |  |
| Acyclovir, Amoxicillin, Amoxicillin/Clavulanate, Ampicillin, Azithromycin, Cefaclor, Cefamandole, Cefazolin, Cefixime, Cefoxitin, Ceftibuten, Ceftriaxone, Cefuroxime, Cephalexin, Cephalothin, Ciprofloxacin, Clarithromycin, Clindamycin, Cloxacillin, Cycloserine, Dirithromycin, Doxycycline, Erythromycin, Ethambutol, Famciclovir, Fluconazole, Gentamicin, Imipenem, Isoniazid, Levofloxacin, Metronidazole, Minocycline, Moxifloxacin, Nitrofurantoin, Norfloxacin, Nystatin, Ofloxacin, Penicillin, Piperacillin, Pivampicillin, Pyrazinamide, Rifampicin, Roxithromycin, Sulfamethoxazole/Trimethoprim, Terbinafine, Tetracycline, Vancomycin | - | 0 |
| ***Antineoplastic and immunomodulating agents*** (N=15) |  |  |
| Anagrelide, Anastrozole, Azathioprine, Bicalutamide, Chlorambucil, Cyclophosphamide, Cyclosporine, Filgrastim, Fluorouracil, Flutamide, Goserelin Acetate, Hydroxyurea, Leuprolide, Methotrexate, Tamoxifen | - | 0 |
| ***Anti-parkinson drugs*** (N=12) |  |  |
| Bromocriptine, Carbegoline, Levodopa/decarboxylase inhibitor, Entacapone, Pramipexole, Ropinirole, Selegiline | - | 0 |
| Amantadine | 2 | 2 |
| Benztropine, Biperiden, Procyclidine, Trihexyphenidyl | 3 | 3 |
| ***Antipsychotics*** *(N=25)* |  |  |
| Lithium, Nemonapride^†^ | - | 0 |
| Blonanserin^†^, Bromperidol^†^, Thiothixene, Ziprasidone | - | 1 |
| Amisulpride, Aripiprazole, Flupentixol, Haloperidol, Paliperidone, Risperidone | 1 | 1 |
| Zotepine^†^ | - | 2 |
| Levomepromazine; Methotrimeprazine, Loxapine, Molindone, Pimozide, Zuclopenthixol | 2 | 2 |
| Perphenazine, Quetiapine | 3 | 2 |
| Chlorprothixene | - | 3 |
| Clozapine, Olanzapine, Thioridazine | 3 | 3 |
| ***Antithrombotic agents*** *(N=6)* |  |  |
| Clopidogrel, Enoxaparin, Heparin, Ticlopidine | - | 0 |
| Dipyridamole, Warfarin | 1 | 0 |
| ***Antivertigo preparations*** *(N=5)* |  |  |
| Betahistine | - | 0 |
| Cinnarizine | 1 | 1 |
| Difenidol | - | 2 |
| Dimenhydrinate, Meclizine | 3 | 3 |
| ***Anxiolytics, hypnotics and sedatives*** *(N=18)* |  |  |
| Buspirone, Chloral hydrate, Clobazam, Melatonin, Nitrazepam, Zolpidem | - | 0 |
| Chlordiazepoxide, Clonazepam, Estazolam, Flunitrazepam, Flurazepam, Lorazepam, Midazolam, Temazepam, Triazolam | - | 1 |
| Alprazolam, Clorazepate, Diazepam | 1 | 1 |
| ***Cardiovascular drugs*** *(N=78)* |  |  |
| Adenosine, Amiloride, Amiodarone, Amlodipine, Atorvastatin, Benazepril, Betaxolol, Bisoprolol, Candesartan, Carvedilol, Cholestyramine, Cilostazol, Clonidine, Diltiazem, Dobutamine, Dopamine, Enalapril, Epinephrine, Eprosartan, Ergoloid mesylates, Ezetimibe, Felodipine, Fenofibrate, Flecainide, Fluvastatin, Fosinopril, Gemfibrozil, Indapamide, Irbesartan, Isradipine, Labetalol, Lercanidipine, Lisinopril, Losartan, Lovastatin, Mannitol, Methyldopa, Metolazone, Mexiletine, Midodrine, Moexipril, Moxonidine, Nisoldipine, Nitroglycerin, Norepinephrine, Olmesartan, Omega-3 triglycerides, Pentoxifylline, Perindopril, Pravastatin, Prazosin, Propafenone, Propranolol, Quinapril, Ramipril, Rosuvastatin, Simvastatin, Sotalol, Spironolactone, Telmisartan, Terazosin, Timolol, Torsemide, Ubidecarenone, Valsartan, Verapamil | - | 0 |
| Atenolol, Captopril, Chlorthalidone, Doxazosin, Hydrochlorothiazide, Isosorbide, Metoprolol, Nifedipine, Triamterene | 1 | 0 |
| Digoxin, Furosemide, Hydralazine | 1 | 1 |
| ***Drug used in diabetes*** *(N=11)* |  |  |
| Acarbose, Glibenclamide, Gliclazide, Glimepiride, Glipizide, Insulin, Metformin, Nateglinide, Pioglitazone, Repaglinide, Rosiglitazone | - | 0 |
| ***Drugs for gastrointestinal conditions and disorders*** *(N=59)* |  |  |
| Aluminium salts, Betaine, Bisacodyl, Caroverine^†^, Casanthranol, Cascara sagrada, Docusate, Domperidone, Esomeprazole, Famotidine, Fenoverine^†^, Frangula, Lactase; Tilactase, Lactic Acid Bacteria, Lactobacillus Rhamnosus, Lactulose, Lansoprazole, Magnesium Preparations, Mesalazine, Metoclopramide, Misiprostol, Nizatidine, Omeprazole, Pancrelipase, Pantoprazole, Papaverine, Pipoxolan^†^, Polycarbophil, Psyllium, Rabeprazole, Senna, Simethicone, Sucralfate, Sulfasalazine, Tiropramide^†^, Ursodiol | - | 0 |
| Alverine | 1 | 0 |
| Trimebutine^†^ | - | 1 |
| Loperamide, Mebeverine, Ranitidine | 1 | 1 |
| Glycopyrrolate | - | 2 |
| Cimetidine, Clidinium | 1 | 2 |
| Cimetropium^†,^ Difemerine^†^, Octylonium bromide^†^, Oxapium iodide^†^ ; Cyclonium, Scopolia extract^†,^ Tiemonium^†^, Timepidium^†^, Tiquizium^†,^ Valethamate bromide^†^ | - | 3 |
| Belladone alkaloids | 2 | 3 |
| Atropine, Dicyclomine ; Dicycloverine, Hyoscyamine, Scopolamine butylbromide, Scopolamine | 3 | 3 |
|  |  |  |
| ***Genitourinary system and sex hormone*** *(N=29)* |  |  |
| Bethanechol, Conjugated Estrogens, Danazol, Dydrogesterone, Esterified Estrogens, Estradiol, Estriol, Estropipate; Estrone Sulfate, Ethinyl Estradiol, Finasteride, Medroxyprogesterone, Megestrol, Methyltestosterone, Norethisterone, Progesterone, Sildenafil, Tadalafil, Tamsulosin, Testosterone, Tibolone, Vardenafil | - | 0 |
| Imidafenacin^†^ | - | 3 |
| Festerodine, Flavoxate, Oxybutynin, Propiverine, Solifenacin, Tolterodine, Trospium | 3 | 3 |
| ***Hormonal preparations*** *(N=14)* |  |  |
| Calcitonin, Carbimazole, Desmopressin, Dexamethasone, Fludrocortisone, Glucagon, Levothyroxine, Liothyronine, Methylprednisolone, Octreotide, Propylthiouracil, Triamcinolone | - | 0 |
| Hydrocortisone, Prednisolone | 1 | 1 |
| ***Mineral and vitamins*** *(N=15)* |  |  |
| Ascorbic acid, Beta-Carotene, Calcitriol, Calcium preparations, Cyanocobalamine, Ergocalciferol, Folic acid, Multivitamin, Niacin, Vitamin K, Potassium, Pyridoxine, Thiamine, Tocopherol, Vitamin E, Zinc preparations | - | 0 |
| ***Musculoskeletal agents*** *(N=21)* |  |  |
| Alendronate, Allopurinol, Chlorzoxazone, Chondroitin, Dantrolene, Eperisone^†^, Etidronate, Glucosamine, Pamidronate, Raloxifene, Risedronate, Succinylcholine, Tolperisone^†^, Vecuronium | - | 0 |
| Colchicine | 1 | 0 |
| Baclofen | 1 | 1 |
| Methocarbamol | 3 | 1 |
| Cyclobenzaprine, Tizanidine | 2 | 2 |
| Pridinol^†^ | - | 3 |
| Orphenadrine | 3 | 3 |
| ***Respiratory agents*** *(N=12)* |  |  |
| Benzonatate, Bromhexine, Montelukast, N-Acetyl-L-Cysteine, Phenylephrine, Pseudoephedrine, Salbutamol; Albuterol, Zafirlukast | - | 0 |
| Dextromethorphan, Guaifenesin | - | 1 |
| Theophylline | 1 | 1 |
| Cloperastine† | - | 2 |
| ***Miscellaneous*** *(N=31)* |  |  |
| Acetazolamide, Acitretin, Activated charcoal, Caffeine, Carnitine, Darbepoetin, Deferasirox, Dextran, Epoetin Alfa, Fish oil, Flumazenil, Ginseng, Glucose, Histidine, Hydroxychloroquine, Influenza Virus Vaccine, Iron preparations, Lidocaine, Methazolamide, Methylcellulose, Methylene blue, Methylphenidate, Modafinil, Naloxone, Pilocarpine, Protamine, Pyridostigmine, Sodium Bicarbonate, Sodium Chloride, Tuberculin Purified Protein Derivate, Varenicline | - | 0 |

† Medications that newly added in K-ABS
