# Supplementary material for: Dose response relationship of cumulative anticholinergic exposure with incident dementia: validation study of Korean anticholinergic burden scale
Source: BMC Geriatr. 2020 Jul 29;20:265. doi: 10.1186/s12877-020-01671-z (PMC7391507; doi:10.1186/s12877-020-01671-z)
Supplement: Supplementary file 2 — Additional file 2. The ICD-10 codes used for analysis. [file 12877_2020_1671_MOESM2_ESM.docx]

**The ICD-10 codes used for analysis**

| **Disease** | **ICD-10 code** |
| --- | --- |
| Alcohol disease | F10, G31.2 |
| Anxiety disorder | F40, F41, F42 |
| Atrial fibrillation | I48 |
| Bipolar disorders | F30, F31, F34.0, F34.8, F34.9 |
| Cerebrovascular disease | G45, G46, I60-I69, H34.0, H34.1, H34.2 |
| Dementia | F00, F01, F02, F03, F051, G30, G31.1 |
| Depression | F32, F33, F34.1 |
| Dyslipidemia | E78 |
| Heart Failure | I11.0, I25.5, I13.0, I13.2, I42, I43, I50 |
| Hypertension | I10–15 |
| Insomnia | G47, F51 |
| Ischemic heart disease | I20-25 |
| Obesity | E66 |
| Parkinson’s disease | G20-22 |
| Schizophrenia | F20-F29 |
| Substance use disorder | F10-F19 |
| Tobacco dependence and tobacco use | F17, Z72.0 |
