# Supplementary material for: Dose response relationship of cumulative anticholinergic exposure with incident dementia: validation study of Korean anticholinergic burden scale
Source: BMC Geriatr. 2020 Jul 29;20:265. doi: 10.1186/s12877-020-01671-z (PMC7391507; doi:10.1186/s12877-020-01671-z)
Supplement: Supplementary file 3 — Additional file 3. List of medication with sedation as a prominent side effects. [file 12877_2020_1671_MOESM3_ESM.docx]

**List of medication with sedation as a prominent side effects**

| **Medication class** | **Ingredients** |
| --- | --- |
| *Primary sedatives, Score 2* | |
| Antidepressants; TCA etc. | Amitriptyline^†^, amoxapine^†^, clomipramine^†^, dothiepin^†^, doxepin^†^, imipramine^†^, mianserine^†^, moclobemide, nortriptyline^†^, quinupramine^†^, trazodone^†^ |
| Barbiturates | Pentobarbital, phenobarbital, thiamylal, thiopental |
| Benzodiazepines | Alprazolam^†^, bromazepam, brotizolam, chlordiazepoxide^†^, clobazam, clonazepam^†^, clorazepate^†^, clotiazepam, diazepam^†^, estazolam^†^, ethyl loflazepate, etizolam, flunitrazepam^†^, flurazepam^†^, lorazepam^†^, mexazolam, midazolam^†^, pinazepam, temazepam^†^, tofisopam, triazolam^†^ |
| General anesthetics | Etomidate, ketamine, propofol |
| Other anxiolytics | Buspirone, hydroxyzine^†^, tandospirone |
| Other hypnotic and sedatives | Chloral hydrate, dichloraphenazone |
| Traditional antipsychotics | Bromperidol^†^, chlorpromazine^†^, chlorprothixene^†^, droperidol, haloperidol^†^, levomepromazine^†^, lithium, mesoridazine^†^, molindone^†^, nemonapride, perphenazine^†^, pimozide^†^, sulpiride^†^, thioridazine^†^, thiothixene^†^, tiapride^†^, trifluoperazine^†^ zotepine^†^, zuclopenthixol^†^ |
| Z-drugs | Zolpidem, zopiclone |
|  |  |
| *Drugs with sedation as a prominent side effect or preparations with a sedating component, Score 1* | |
| Antidepressants; SSRI etc. | Bupropion^†^, citalopram^†^, duloxetine, escitalopram^†^, fluoxetine^†^, fluvoxamine^†^, hyperici herba, medifoxamine fumarate, milnacipran, mirtazapine^†^, nefazodone, paroxetine^†^, sertraline, tianeptine, toloxatone, venlafaxine^†^ |
| Antiepileptics | Carbamazepine^†^, fosphenytoin, gabapentin, lamotrigine, levetiracetam, oxcarbazepine^†^, phenytoin, pregabalin, primidone, topiramate, valproic acid, vigabatrin, zonisamide |
| Antimigraine | Almotriptan, ergotamine, frovatriptan, naratriptan, sumatriptan, zolmitriptan |
| Antiparkinson agents | Benzatropine^†^, biperiden^†^, procyclidine^†^, trihexyphenidyl^†^ |
| Antivertigo & antiemetics | Betahistine, cinnarizine^†^, difenidol^†^, dimenhydrinate^†^ |
| Atypical antipsychotics | Amisulpride^†^, aripiprazole^†^, blonanserin^†^, clozapine^†^, olanzapine^†^, paliperidone^†^, quetiapine^†^, risperidone^†^, ziprasidone^†^ |
| Central muscle relaxants | Afloqualone, baclofen^†^, carisoprodol^†^, chlormezanone, chlorphenesin, chlorzoxazone, cyclobenzaprine^†^, eperisone, methocarbamol^†^, orphenadrine^†^, pridinol^†^, thiocolchicoside, tizanidine^†^, tolperisone |
| Opioids | Alfentanil, buprenorphine, butorphanol, codeine^†^, dihydrocodeine, fentanyl^†^, hydrocodone^†^, hydromorphone, morphine^†^, nalbuphine, oxycodone^†^, oxycodone and naloxone^†^, pentazocine, pethidine^†^, remifentanil, sufentanil, tramadol^†^ |
| Old antihistamines^†^ | Alimemazine, brompheniramine, buclizine, carbinoxamine, chlorcinnazine, chlorpheniramine, clemastine, cyproheptadine, dexbrompheniramine, diphenhydramine, diphenylpyraline, doxylamine, homochlorcyclizine, mequitazine, oxatomide, oxomemazine, pheniramine, piprinhydrinate, triprolidine, promethazine |
| Prokinetics | Metoclopramide |
| Respiratory | Benproperine, benzonatate, bromhexine, dextromethorphan^†^, levodropropizine |

^†^ Medications with anticholinergic properties which were excluded when measuring the sedative load in this current study
